# The Involvement of Microtubules and Actin during the Infection of Japanese Encephalitis Virus in Neuroblastoma Cell Line, IMR32

**DOI:** 10.1155/2015/695283

**Published:** 2015-02-01

**Authors:** Magdline Sia Henry Sum

**Affiliations:** Institute of Health and Community Medicine, Universiti Malaysia Sarawak, 94300 Kota Samarahan, Sarawak, Malaysia

## Abstract

The role of the cytoskeleton, actin, and microtubules were examined during the process of Japanese encephalitis (JEV) infection in a human neuroblastoma cell line, IMR32. Cytochalasin D and nocodazole were used to depolymerise the cellular actin and microtubules, respectively, in order to study the effect of JEV infection in the cell. This study shows that depolymerisation of the actin cytoskeleton at early process of infection inhibits JEV infection in the cell; however infection was not inhibited when depolymerisation occurred at the later stage of infection. The microtubules, on the other hand, are required at 2 points in infection. The antigen production in the cells was inhibited when the infected cells were treated at time up to 2 hours after inoculation and there was no significant effect at later times, while the viable virus released continued to be affected until 10 hours after inoculation. In conclusion, infection of JEV in IMR32 cells required actin to facilitate early process in infection and the microtubular network is utilised as the transport system to the virus replication site and the release of mature virus.

## 1. Introduction

Japanese encephalitis virus (JEV), a member of the family* Flaviviridae*, is a major cause of virus encephalitis in Asia. Swine are the amplifying hosts of JEV and the virus is transmitted in a zoonotic cycle by mosquitoes to some wild and domestic animals such as swine and wading birds and to humans who are incidental hosts. Most JEV infections of humans are asymptomatic and only a small proportion of them progress to encephalitis, but when they do, the mortality is high and survivors often develop serious neurological sequelae [1]. JEV is an enveloped virus that contains a single stranded positive sense RNA genome that is approximately 11 kb in length. The genome contains a single open reading frame encoding three structural proteins—the capsid (C), premembrane/membrane (prM/M) and envelope (E) protein, and seven nonstructural proteins—and it gives its name to the Japanese encephalitis virus serocomplex to which West Nile virus (WNV), Murray Valley encephalitis virus (MVEV), St. Louis encephalitis virus (SLEV), and Kunjin virus (KUNV) also belong [2].

The process of infection of mammalian and mosquito cells by JEV has been much less investigated than that of other viruses of this serocomplex, especially WNV. Like other flaviviruses, it is known that JEV utilises a clathrin-dependent endocytic route of entry and exit from endosomes requires a drop in pH [3–5]. WNV also uses the endosomal pathway and the use of Eps15 dominant negative mutants and clathrin-blocking drugs has demonstrated the requirement for clathrin in this process for both mammalian and mosquito cells [6, 7]. Disruption of the microtubule network affects trafficking of WNV structural proteins in the infected cell [8] and it has been suggested that the actin cytoskeleton is necessary for the entry of WNV into mammalian cells [7] but not mosquito cells [6]. An intact actin cytoskeleton has also been shown to be required for the release of mature WNV virions [9].

Many viruses use the cytoskeleton network of the cell as a transport system and as a guide to the site of virus replication in the cell. Adenovirus particles have been shown in association with microtubules by electron microscopy as early as the 1970s [10] and it is also shown that retrograde transport of some viruses such as HSV1 involves both microtubules and motors associated with microtubules as reviewed by Ploubidou and Way [11]. Actin has been implicated in movements closer to the plasma membrane such as entry and exit of viruses [11].

This study investigated the involvement of the cytoskeleton actin and microtubules in cellular transport of JEV by studying the effect of the cytoskeleton-disrupting drugs nocodazole and cytochalasin D, and this study also reports the dynamics of the contribution of actin and microtubules towards a productive JEV infection of the human neuroblastoma cells, IMR32.

## 2. Materials and Methods

### 2.1. Drug and Antibodies

Nocodazole and cytochalasin D were purchased from Sigma (St. Louis, MI, USA). Primary antibodies used were mouse anti-MAP2b and mouse anti-EEA1 from BD Biosciences (USA), mouse anti-*β* tubulin-1 and mouse anti-golgi 58 from Sigma (St. Louis, MI, USA), and mouse anti-ER from Molecular Probes (Eugene, OR, USA). Phalloidin BODIPY 558/568 and secondary antibodies anti-mouse isotype specific (IgG2a) Alexa Fluor 488, anti-mouse isotype specific (IgG1) Alexa Fluor 594, and anti-mouse isotype specific (IgG2b) Alexa Fluor 594 were all purchased from Molecular Probes (Eugene, OR, USA). Mouse monoclonal antibodies, MV12/1/C2-2/1 (IgG2a) and MV12/2/A5-1/5 (IgG3) specific for JEV E and NS1, respectively, were supplied by Venture Technologies (Malaysia). The flavivirus reactive mouse monoclonal antibody 4G2 was produced in-house. Anti-human IgG HRP and anti-mouse IgG HRP were purchased from DAKO Cytomation (Glostrup, Denmark).

### 2.2. Cell Lines and Virus Strains

Viruses were propagated in C6/36* Aedes albopictus* cells, in a growth medium of Leibovitz 15 supplemented with 3% heat inactivated fetal bovine serum, 10% tryptose phosphate broth, 50 U/mL penicillin G, and 50 *μ*g/mL streptomycin sulphate. All media components were purchased from Life Technologies (Gaithersburg, MD, USA). Viruses used were JEV prototype Nakayama and a local isolate JEV CNS138/9. Unless stated otherwise, all immunofluorescence and infectivity studies were done using the neuroblastoma cell line, IMR32 which was maintained in Dulbecco's modified Eagle's medium (DMEM), supplemented with 10% heat inactivated fetal bovine serum, 50 U/mL penicillin G, and 50 *μ*g/mL streptomycin sulphate. Plaque assays were performed in PS Clone D, a continuous porcine kidney cell line grown in Leibovitz 15 supplemented with 3% heat inactivated fetal bovine serum, 10% tryptose phosphate broth, 50 U/mL penicillin G, and 50 *μ*g/mL streptomycin sulphate.

### 2.3. Immunofluorescence Microscopy

Experiments were performed using cells adhered overnight to wells of 8- or 10-well multitest slides (Cel-Line slides, Erie Scientific Co., Portsmouth, NH, USA). Cells were treated with either cytochalasin D or nocodazole at 0.5 *μ*g/mL and 10 *μ*g/mL, respectively, or otherwise as stated. Treated and untreated cells were infected with JEV at a MOI of 1. The slides were washed with PBS before fixing with ice-cold acetone for 10 minutes. The cells were blocked for 30 minutes with PBS/5% BSA. All washing steps were done using PBS/1% BSA for 3 times at 10-minute interval. The acetone fixed slides were stained by incubating for 30 minutes with primary antibody followed by 30 minutes with the secondary antibody and mounted with ProLong Gold containing DAPI purchased from Molecular Probes (Eugene, OR, USA). All incubation and washing steps were performed at room temperature. Slides were viewed using an Axiovert 200 (Zeiss, Germany) with filter sets appropriate for FITC, Rhodamine, and DAPI. Photomicrography was achieved using a cooled CCD monochrome 12 bit camera Evolution QEi and Image-Pro 5.0 software (Media Cybernetics Inc., Canada) was used for preparing fluorescence composite images with pseudocolour. Adobe Photoshop version 5.0 LE was used to compose and present the figure collages.

### 2.4. Drug Treatment

IMR32 cells were treated with either cytochalasin D or nocodazole at different concentrations in the dose response experiments or otherwise at 0.5 *μ*g/mL and 10 *μ*g/mL, respectively. The cells were inoculated with JEV at MOI of 1 for 30 minutes at 37°C. The inoculum was then removed and the cell monolayers were washed twice with PBS. The cells were then replenished with either media only or media containing the inhibitors and remained throughout the experiment. The supernatants were harvested after 72 hours and the amount of virus produced was determined in a plaque assay as well as in an antigen capture ELISA for new virus progeny and antigen production, respectively. All experiments using drug treatment, mock treatment with the equivalent solvent (DMSO) dilution were used as controls, to eliminate solvent effects in our results.

### 2.5. Plaque Assay

PS Clone D cells were suspended at a density of 3 × 10^5^ cells/mL and 0.5 mL was dispensed in each well of 24-well plates. Tenfold serial dilutions were prepared from supernatants harvested from individual wells of the infectivity experiments. One hundred *μ*L of each dilution was added into the wells and incubated for 37°C for 3 hours before adding 0.5 mL semisolid overlay containing 1% carboxymethylcellulose. Cell monolayers were stained after 3 days with naphthalene black for 20 minutes before rinsing off with water. Plaques were counted to determine the number of plaque forming units (pfu) per mL of harvested supernatant.

### 2.6. Fluorescence Focus Assay

The assay was performed using 8-well multitest slides (CEL-LINE slides, Eerie Scientific Co., Portsmouth, NH, USA). Cells were seeded on the wells at 1 × 10^5^ cells/mL at 30 *μ*L/well and left to adhere overnight in a moist chamber in a 37°C incubator supplemented with 5% CO_2_. The cell monolayers were infected with JEV at MOI of approximately one. The cells were either treated with cytochalasin D or the DMSO control 30 minutes prior to infection. Cells were acetone fixed 16 hours after infection. The cells were stained with MV12/1/C2-2/1, monoclonal antibody specific for E of JEV. Images of four independent fields of each well were captured and the numbers of infected cells from each of the four fields were counted. The total number of cells in each field was determined by the number of intact DAPI-stained nuclei counted.

### 2.7. Antigen Capture ELISA

96-well flat-bottomed ELISA plates (Maxisorb, Nunc, Denmark) were coated with anti-mouse immunoglobulin (DAKO, Denmark) at 1 : 2000 dilution in carbonate-bicarbonate coating buffer, pH 9.6 overnight at 4°C. The wells were blocked with 1% casein in PBS followed by an overnight incubation with MV12/1/C2-2/1 (Venture Technologies, Malaysia), a monoclonal antibody specific for the envelope protein of JEV. Supernatants harvested from the infectivity assays were incubated overnight in duplicate wells followed by incubation with a high titred human convalescent flavivirus serum, PPCS (Venture Technologies, Malaysia) for one hour. Bound antigen was detected using anti-human IgG HRP (DAKO, Denmark) and colour was developed using a chromogenic substrate containing OPD and hydrogen peroxide. The absorbance was measured in a plate reader Elx50 (Bio-Tek Instruments Inc., USA) and read at dual wavelengths 492 and 650 nm.

## 3. Results

### 3.1. Time Course Study of JEV Infection in IMR32

**Figure 1:**
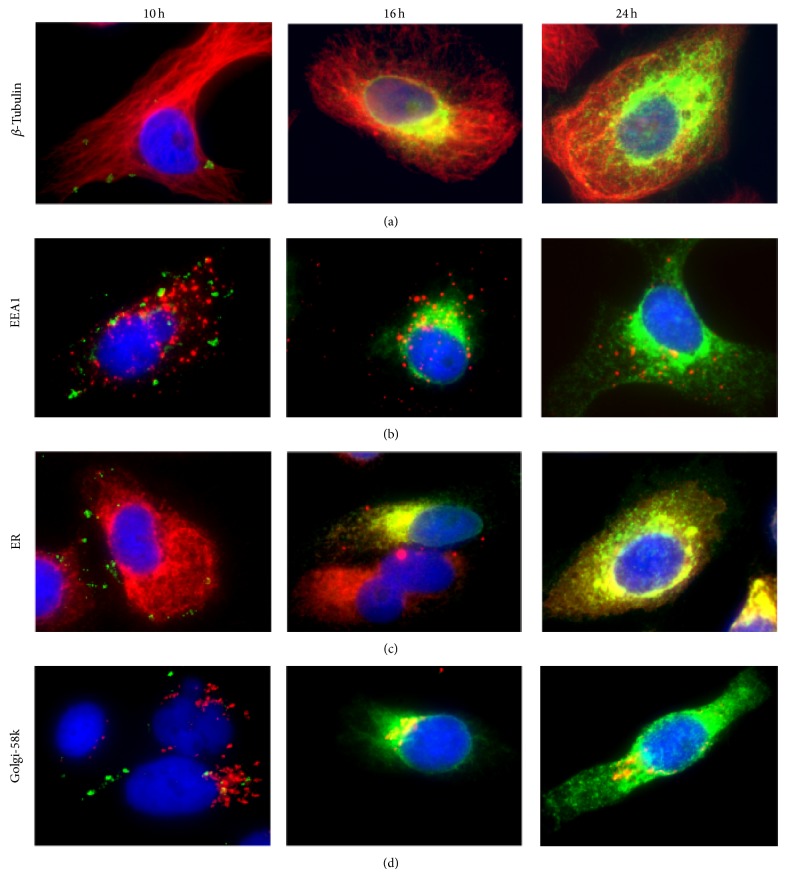
Cells were fixed at 10 h, 16 h, and 24 h after infection. Staining was done to detect the presence of E (detected by MV12/1/C2-2/1) antigen (green) in relation to cellular proteins (red) (a) *β*-tubulin, (b) EEA1, (c) ER, and (d) Golgi-58k as presented in the different rows above.

**Figure 2:**
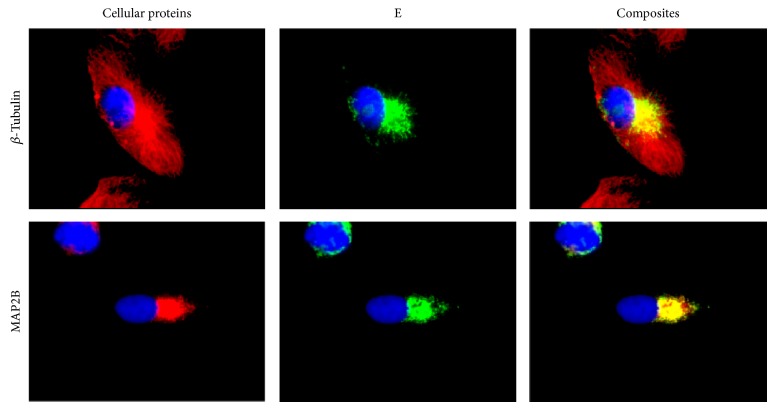
Colocalization of E at the MTOC. Dual labelling of the JEV E protein with *β*-tubulin and MAP2B protein showing the colocalization of the E protein at the MTOC of the cells at 16 h p.i. The cellular protein was detected using goat anti-mouse isotype specific antibody (IgG1) conjugated with Alexa Fluor 594 (red) and E with goat anti-mouse isotype specific antibody (IgG2a) conjugated with Alexa Fluor 488 (green). Cells were counterstained with DAPI (blue).

In a time course study, JEV infected IMR32 cells were fixed at 10, 16, and 24 hours after infection. The E antigen of JEV was detected by specific monoclonal antibody to E MV12/1/C2-2/1. Small packets of antigen were first seen scattered around the cell, mainly in the periphery at 10 hours after infection (Figure 1, first column). Antigen distribution was later seen to be concentrated in the perinuclear region at 16 hours after infection (Figure 1, second column) and by 24 hours had spread from the perinuclear region through the cytoplasm and towards the cell periphery (Figure 1, third column). In Figure 1, dual staining of infected cells for JEV E and different cell structures, microtubules stained with anti-*β* tubulin, early endosomes stained with anti-EEA1, endoplasmic reticulum stained with anti-ER, and Golgi stained with antibodies to the 58 K protein are also shown. The spread of the viral antigen to the periphery of the infected cells is most clearly seen in the series stained for microtubules (Figure 1(a)). The figure shows that at 16 hours (Figure 1(a)) and 24 hours (Figure 1(c)), the E antigen of JEV show co-localization with microtubules and the endoplasmic reticulum. At 16 hours the presence of E was also detected in the Golgi but by 24 hours much of the antigen had left this compartment although there was still Golgi associated E (Figure 1(d)). By 10 hours after infection there was no appreciable colocalization of viral antigen with endosomes as seen in Figure 1(b).  Figure 2 shows cells fixed at 16 h after infection with clear localization of the JEV E with the microtubule organizing centre region (MTOC) as detected by dual staining using either the JEV E specific monoclonal antibody, MV12/1/C2-2/1 with antibodies against *β* tubulin protein, a marker for the MTOC.

### 3.2. Actin Facilitates Successful JEV Infection of Cells

**Figure 3:**
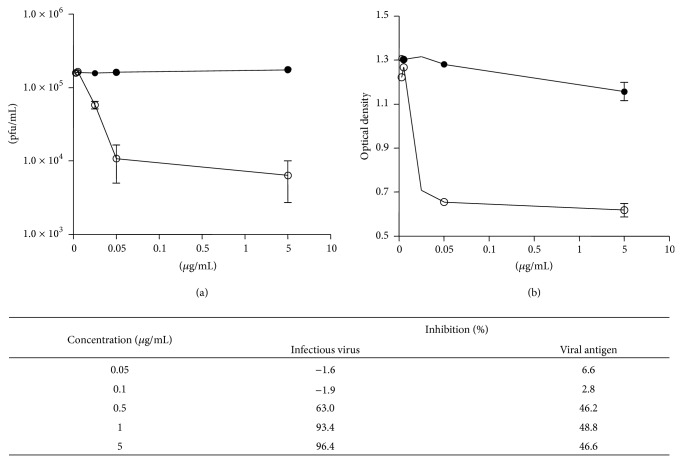
Dose response curves for cytochalasin D showing the effect of the drug on production of infectious virus (a) and on viral antigen (b). Cells were treated with drug (open circles) or the DMSO control (close circles) and culture supernatants were harvested after 72 hours and tested for infectious virus (pfu/mL) or for antigen (OD). Experiments were done in duplicate, with duplicate data points in each experiment.

IMR32 cells infected with JEV were treated with cytochalasin D at 30 minutes prior to inoculation of virus at 5 different drug concentrations between 0.05, 0.1, 0.5, 1, and 5 *μ*g/mL. The effect of cytochalasin D on JEV infection in the cells was measured by quantifying infectious virus and viral antigen produced at 72 hours after infection. The percentage of inhibition of infectious virus and virus antigens at different concentrations of cytochalasin D is shown in Figure 3. There was almost complete inhibition of infectious virus production at 1 and 5 *μ*g/mL. However, at doses of cytochalasin D of 1 *μ*g/mL or more, the cells displayed effects of toxicity while at 0.5 *μ*g/mL, the cells were still intact although disassembly of the actin filaments was evident. Therefore for the subsequent experiments 0.5 *μ*g/mL cytochalasin D was used because at higher doses it was unable to distinguish between inhibitions of infectious virus production due to the drug or due to cytotoxicity. Virus antigen was reduced by 46% to 49% at 0.5 *μ*g/mL or more of cytochalasin D. At doses lower than 0.5 *μ*g/mL, there was no appreciable inhibition of virus antigen production.

**Figure 4:**
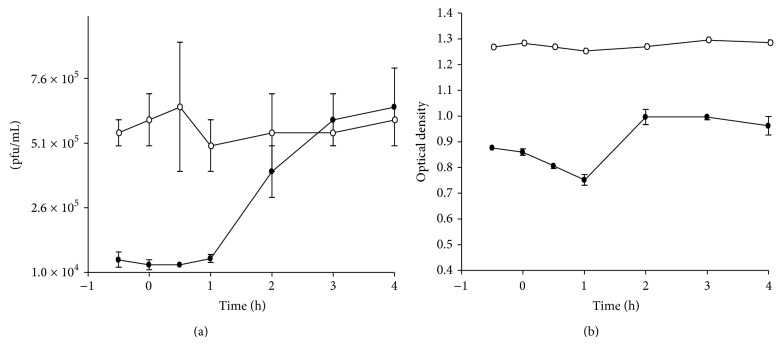
Time course of treatment of cells with cytochalasin D. The effect of cytochalasin D (0.5 *μ*g/mL) treatment on cells at different times in relation to infection was determined by measuring infectious virus and viral antigen in supernatants harvested at 72 hours. (a) Effect on infectious virus production. (b) Effect on viral antigen. The closed circles represent the drug and the open circles the DMSO control. Each data point was done in quadruplicate.

In order to determine when intact actin filaments were necessary for the production of antigen and the release of viable virus, IMR32 cells were treated with cytochalasin D at various time points relative to inoculation of virus and harvested the culture supernatant at 72 hours after infection. Viral antigen and viable virus were measured in antigen capture ELISAs and plaque assays, respectively.  Figure 4(a) shows that the amount of viable virus released was reduced by about 1 log when the cells were treated at 30 minutes prior to inoculation, at the time of inoculation and up to 1 hour after inoculation. When cytochalasin D was added to cells at 3 hours or later after infection, there was no effect on the production of viable virus. A similar trend was also observed when virus antigen was measured (Figure 4(b)).

### 3.3. Depolymerization of Microtubules Reduces Virus Infectivity

**Figure 5:**
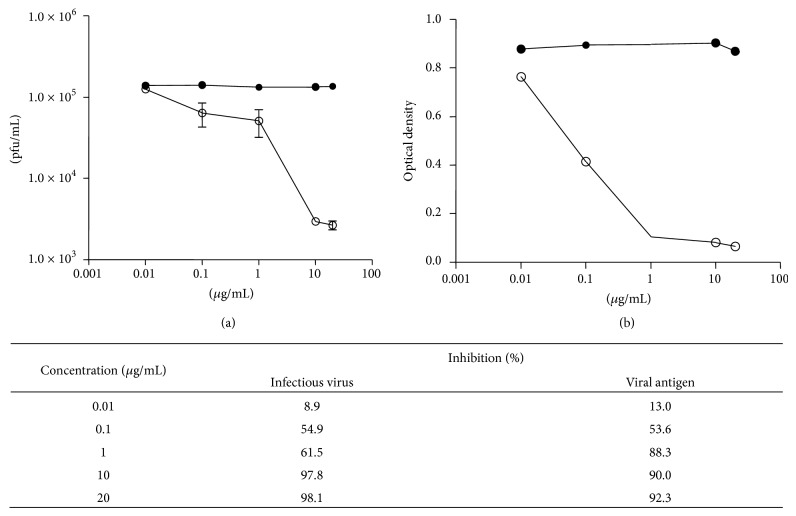
Dose response curves for nocodazole showing the effect of the drug on production of infectious virus (a) and on viral antigen (b). Cells were treated with drug (open circles) or the DMSO control (close circles) and culture supernatants were harvested after 72 hours and tested for infectious virus (pfu/mL) or for antigen (OD). Experiments were done in duplicate, with duplicate data points in each experiment.

IMR32 cells were treated with 0.01, 0.1, 1, 10, and 20 *μ*g/mL of nocodazole and it was found that the titre of viable virus released as well as the amount of viral antigen produced was reduced in a dose dependent manner.  Figure 5 shows the percentage of inhibition of antigen and infectious virus at the different doses of nocodazole. The extent of inhibition of both antigen and infectious virus was considerably 90–100% for infectious virus and about 80% for viral antigen.

**Figure 6:**
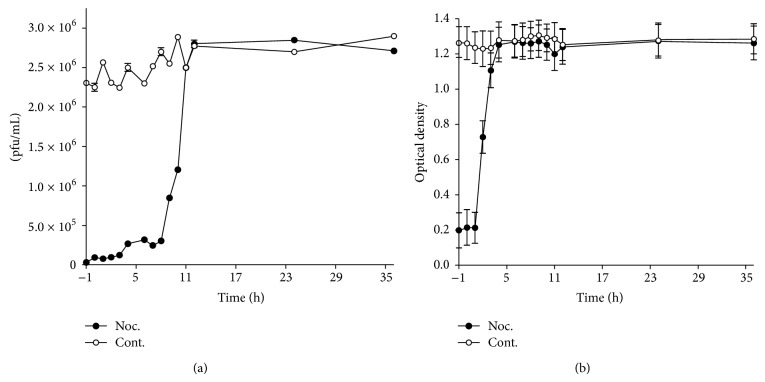
Time course of treatment of cells with nocodazole. Medium containing 10 *μ*g/mL nocodazole was added to the cells at the indicated time points relative to infection and maintained for the entire duration of the 72 h incubation. Control cells were incubated with medium containing the equivalent amount of the solvent DMSO. Infectious virus (a) and viral antigen (b) were measured. The closed circles represent the drug and the open circles the DMSO control.

To determine when during the infection process microtubules were required, cells were treated with 10 *μ*g/mL nocodazole in a time series from 1 hour prior to inoculation to 36 hours after inoculation with virus. For each treatment time point, cell culture supernatant was harvested 72 hr after inoculation and evaluated for release of viable virus and antigen production. The result of the ELISA was affected only if cells were treated early (Figure 6(b)) but the plaque assay result showed reduction even when nocodazole was added to the cells hours after inoculation (Figure 6(a)). This result showed that even though virus assembly occurs, the viral particles lack infectivity.

## 4. Discussion and Conclusion

Studies with the related flavivirus WNV have shown that WNV requires actin for entry into Vero cells, and microtubules have a role in trafficking virus from early endosomes to lysosomes, but the kinetics of the infection process by WNV is considerably faster than other flaviviruses, with a latent period between 4 and 6 hours as compared to 16 and 24 hours for other flaviviruses [7]. JEV clearly falls into the group of flaviviruses with a longer latent period. We show that both actin filaments and microtubules are needed in the course of JEV infection of neuroblastoma cells. The cytoplasm is highly viscous and many viruses are known to use the dynamic actin network to traverse the plasma membrane and the actin cortex of the cell [12]. For this study cytochalasin D and nocodazole were used to depolymerize actin and the microtubules, respectively, as shown (see Supplementary Figure 1 in the Supplementary Material available online at http://dx.doi.org/10.1155/2014/695283). The concentration of both cytochalasin D and nocodazole was optimized to show the best result without affecting the cell viability; for this study cytochalasin D and nocodazole were used at 0.5 *μ*g/mL and 10 *μ*g/mL, respectively (Supplementary Figure 2). The data from this study suggests that actin filaments are involved early in the process of JEV infection of neuroblastoma cells and this early event takes place in the first 30 minutes after inoculation. The action of actin takes place mainly at the periphery of the cell, and we have shown using a fluorescence focus assay that the disruption of the actin cytoskeleton causes a reduction in the number of cells successfully infected by JEV. We also show that the production of infectious virus requires a dynamic actin cytoskeleton up to around 2 hours after infection. After the actin dependent entry of JEV, transport to the MTOC is mediated by microtubules. When the microtubular network is disrupted by nocodazole up to 2 hours after inoculation, the significant reduction in antigen production ensues likely because the depolymerization of the microtubules prevents the infecting virus from reaching the MTOC and sites of viral replication. Disruption of the microtubular network later than 2 hours after infection does not affect the production of antigen but inhibits the production of viable virus even when nocodazole is added many hours later, suggesting that microtubules play a role at 2 points in the process of JEV infection during antigen production and for viable virus release. This result suggests that microtubules are required for JEV infection first during retrograde transport along microtubules to the MTOC where replication takes place and second during antiretrograde transport when assembly of mature virus particles is taking place. In conclusion the results of this study are consistent with JEV using actin early in infection to overcome the barrier of the plasma membrane. The results of this study also suggest that JEV utilizes the microtubules highway to assist in the trafficking of the viral particles and this result is consistent with study on WNV by Chu and Ng (2002) [8]. The two time points' requirement shown in this study may suggest the involvement during viral replication as well as release of new virus progeny. Further investigations into the kinetics and the motors that JEV cargo utilises at the various stages of the infection process are underway.

## Supplementary Material

The images in the supplementary material show the effect of cytochalasin D (supplementary Figure 1) and nocodazole (supplementary Figure 2) on the IMR32 cells, infected with JEV. The cells were treated with cytochalasin D and nocodazole at 0.5 ωg/ml and 10 µg/ml respectively in separate experiments. Since both cytochalasin D and nocodazole were reconstituted in DMSO, similar experiments with cells treated with DMSO alone were conducted as control. In supplementary Figure 1, cells were fixed at 72 h post infection and JEV E antigen was stained with monoclonal antibody MV12/1/C2-2/1 (green), actin was detected by staining with phalloidin BODIPY 588/568 (red). In supplementary Figure 2, cells were fixed at 72 h post infection and JEV E antigen was stained with monoclonal antibody MV12/1/C2-2/1 (top row) while JEV NS1 antigen was stained with monoclonal antibody MV12/2/A5-1/5 (bottom row). In both experiments, nuclei were stained with DAPI (blue) to show the viability of the cells.
