# Supplementary material for: The Involvement of Microtubules and Actin during the Infection of Japanese Encephalitis Virus in Neuroblastoma Cell Line, IMR32
Source: Biomed Res Int. 2015 Feb 1;2015:695283. doi: 10.1155/2015/695283 (PMC4331156; doi:10.1155/2015/695283)
Supplement: Supplementary file 1 — The images in the supplementary material show the effect of cytochalasin D (supplementary Figure 1) and nocodazole (supplementary Figure 2) on the IMR32 cells, infected with JEV. The cells were treated with cytochalasin D and nocodazole at 0.5 ωg/ml and 10 µg/ml respectively in separate experiments. Since both cytochalasin D and nocodazole were reconstituted in DMSO, similar experiments with cells treated with DMSO alone were conducted as control. In supplementary Figure 1, cells were fixed at 72 h post infection and JEV E antigen was stained with monoclonal antibody MV12/1/C2-2/1 (green), actin was detected by staining with phalloidin BODIPY 588/568 (red). In supplementary Figure 2, cells were fixed at 72 h post infection and JEV E antigen was stained with monoclonal antibody MV12/1/C2-2/1 (top row) while JEV NS1 antigen was stained with monoclonal antibody MV12/2/A5-1/5 (bottom row). In both experiments, nuclei were stained with DAPI (blue) to show the viability of the cells. [file 695283.f1.pdf]

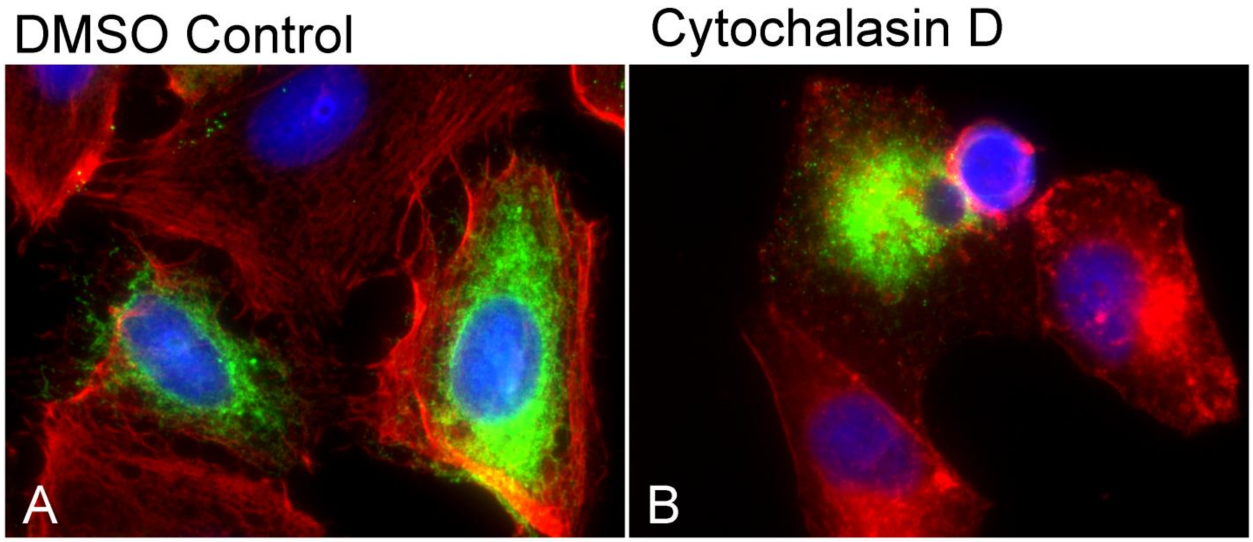

Supplementary Figure 1. IMR32 cells infected with JEV in the presence of cytochalasin D or the equivalent DMSO control. Infected cells treated with DMSO (A) or 0.5µg/ml cytochalasin D (B) upon inoculation and maintained for the entire duration. Cells were fixed at 72 h p.i and JEV E antigen was stained with monoclonal antibody MV12/1/C2-2/1 (green), actin was detected by staining with phalloidin BODIPY 588/568 (red) and the nuclei were stained with DAPI (blue) to show the viability of the cells.

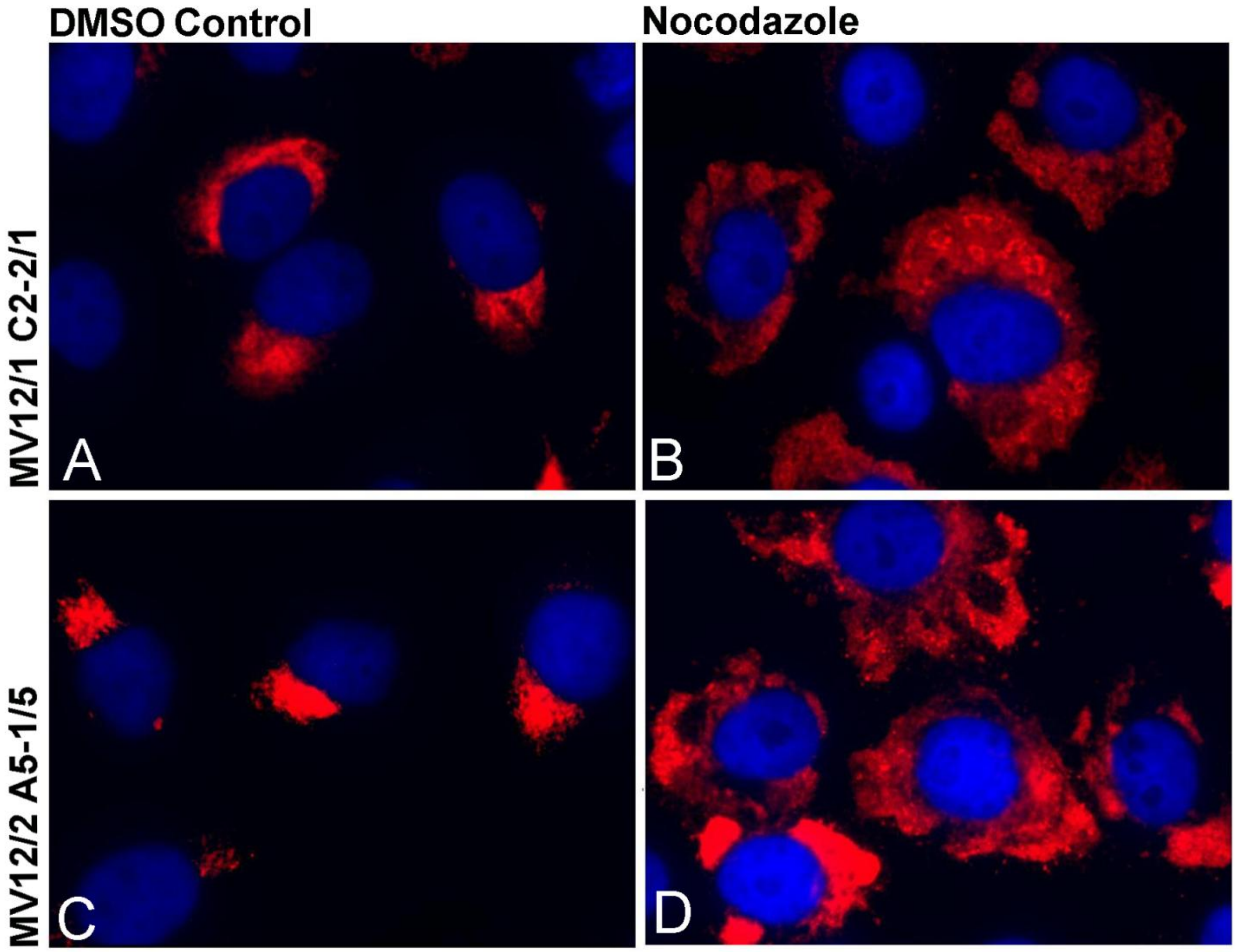

Supplementary Figure 2. IMR32 cells infected with JEV in the presence of nocodazole or the equivalent DMSO control. Infected cells were treated with DMSO as control (A and C) or 10µg/ml nocodazole (B and D) upon inoculation and maintained for the entire duration. Cells were fixed at 72 h p.i and JEV E antigen was stained with monoclonal antibody MV12/1/C2-2/1 (top row) while JEV NS1 antigen was stained with monoclonal antibody MV12/2/A5-1/5 (bottom row). Nuclei were stained with DAPI (blue) to show the viability of the cells.
